# Microstimulation reveals anesthetic state-dependent effective connectivity of neurons in cerebral cortex

**DOI:** 10.3389/fnins.2024.1387098

**Published:** 2024-07-05

**Authors:** Anthony G Hudetz

**Affiliations:** Department of Anesthesiology, Center for Consciousness Science, University of Michigan, Ann Arbor, MI, United States

**Keywords:** microstimulation, cortex, network, connectivity, spike, anesthesia, consciousness

## Abstract

**Introduction:**

Complex neuronal interactions underlie cortical information processing that can be compromised in altered states of consciousness. Here intracortical microstimulation was applied to investigate anesthetic state-dependent effective connectivity of neurons in rat visual cortex *in vivo*.

**Methods:**

Extracellular activity was recorded at 32 sites in layers 5/6 while stimulating with charge-balanced discrete pulses at each electrode in random order. The same stimulation pattern was applied at three levels of anesthesia with desflurane and in wakefulness. Spikes were sorted and classified by their waveform features as putative excitatory and inhibitory neurons. Network motifs were identified in graphs of effective connectivity constructed from monosynaptic cross-correlograms.

**Results:**

Microstimulation caused early (<10 ms) increase followed by prolonged (11–100 ms) decrease in spiking of all neurons throughout the electrode array. The early response of excitatory but not inhibitory neurons decayed rapidly with distance from the stimulation site over 1 mm. Effective connectivity of neurons with significant stimulus response was dense in wakefulness and sparse under anesthesia. The number of network motifs, especially those of higher order, increased rapidly as the anesthesia was withdrawn indicating a substantial increase in network connectivity as the animals woke up.

**Conclusion:**

The results illuminate the impact of anesthesia on functional integrity of local cortical circuits affecting the state of consciousness.

## Introduction

Complex neuronal interactions underlie cortical information processing from which cognitive functions and consciousness emerge. Anesthetic agents are unique tools to alter neuronal behavior with the goal to better understand the neurobiological basis of normal and altered states of consciousness. Most prior work examined changes in neuronal connectivity through recordings of spontaneous ongoing activity or sensory stimulus-evoked activity during different wakeful and anesthetized states (Hudetz et al., 2009; Andrada et al., 2012; Vizuete et al., 2012; Aasebo et al., 2017; Lee et al., 2021; Bharioke et al., 2022; Margalit et al., 2022; Aggarwal et al., 2023). However, recent studies suggest that further insight into the functional interaction of neurons may be gained through exogenous perturbation of single neurons or local neuronal populations.

Former studies proposed to employ intracortical microstimulation to probe the structure and function of intact neuronal circuits (Clark et al., 2011; Kwan and Dan, 2012; Kumar et al., 2013). This approach can help reveal the causal or “effective” connectivity of neurons (Aertsen et al., 1989) that is not directly discoverable from the recording of undisturbed, spontaneous activity alone (Clark et al., 2011; Kumar et al., 2013; Sadeh and Clopath, 2020). Microstimulation also helps mitigate the confounding effect of background activity of other neurons, in analogy with what is achieved by evoked potential averaging (Cheney et al., 2013). The effect of anatomically precise perturbation by microstimulation can propagate throughout the neuron network, generating multiple extra spikes that last for hundreds of ms (London et al., 2010; Kwan and Dan, 2012; Emiliani et al., 2015; Bernardi et al., 2021). Such spike sequences formed by forward and recurrent interactions may reflect basic information packets in the neuron network (Luczak et al., 2015). Anesthetics can also alter the spike sequences (Tanabe et al., 2023), which could inform the potential mechanism by which state-dependent cellular changes may impair neuronal information processing and consciousness (Hudetz et al., 2015; Margalit et al., 2022; Pazienti et al., 2022).

Intracortical stimulation was recently used to examine the effects of anesthesia on electroencephalographic connectivity (Arena et al., 2021) but not the connectivity of discrete neurons in local cortical circuits. To fill this gap of knowledge, this work applied intracortical microstimulation to investigate how anesthesia achieved with the inhalational agent desflurane may alter stimulus-related spiking activity and effective connectivity of discrete neurons in rat visual cortex *in vivo*. By electrically stimulating neuronal tissue at one of the sites of an intracortical microelectrode array and measuring the spiking activity of neurons at all other electrode sites, the effective connectivity of the neuronal network was estimated. We hypothesized that the anesthetic would disrupt effective connectivity of the neurons in a dose-dependent manner, implying a gradual disruption of information processing. We also examined how the anesthetic may influence the stimulus response of putative excitatory vs. inhibitory neurons. Most inhalational anesthetics suppress spontaneous neuronal activity by facilitating inhibitory synaptic transmission and suppressing excitatory synaptic transmission (Franks, 2008), but the state-dependent effects of intracortical microstimulation on excitatory vs. inhibitory neuron firing are unclear. We anticipated that the effects of anesthesia on microstimulation-induced neuron firing will also be cell type dependent. Overall, the study aimed to gain insight into how anesthesia impacts the functional integrity of cortical neuronal connectivity relevant for the loss or return of consciousness.

## Materials and methods

### Surgery and experimental protocol

The study was approved by the Institutional Animal Care and Use Committee in accordance with the Guide for the Care and Use of Laboratory Animals of the Governing Board of the National Research Council (National Academies of Sciences, Engineering, and Medicine, 2011). General procedures followed those described before (Lee et al., 2020) but using a different probe design. Briefly, nine adult male and female rats were chronically implanted with microelectrode arrays (Microprobes, Gaithersburg, MD, USA) into the right primary visual cortex (V1) for extracellular recording and stimulation. The probes consisted of 32 platinum/iridium (70%/30%) electrodes of 75 μm diameter and 2 mm length. They had approximately 100 kOhm impedance. The electrodes were arranged in a 6 × 6 square matrix format, omitting the corner locations. The electrode spacing was 250 μm in both directions. The stereotaxic target coordinates of the center of the array at the electrode tips were −6.75 mm anteroposterior, 3.60 mm mediolateral, and 1.50 mm deep from Bregma. The depth was chosen to target infragranular cortex (layers 5/6); this was not verified with histological analysis in this study. One corner of the electrode array was occupied by the reference electrode consisting of the same Pt/Ir composition with 10 kOhm impedance. An additional low-impedance electrode placed in the opposite corner of the array or an external bare wire tied to a stainless-steel screw in the cranium over the left cerebellum served as ground.

Several days after recovery the animals were placed unrestrained in a sealed, dark anesthesia chamber for testing. The experiment consisted of stepwise decrease of inhaled concentration of desflurane at 6, 4, 2.5 and 0% mixed to 30% oxygen-enriched air. Desflurane concentrations were chosen to cover a suitable range from light sedation to deep anesthesia. Based on our former experiments (Imas et al., 2005) with testing the righting reflex–a putative index of consciousness in rodents (Franks, 2008), 2.5% corresponds to conscious sedation, 6% unconsciousness and 4% is near the transition point. Anesthetic depth was not tested in the present experiments. Each anesthetic concentration was held for 60 min including an equilibration time of 15 min before commencing microstimulation. A typical experiment lasted for 4 h.

### Stimulation parameters

Monopolar stimuli with respect to ground were delivered to each electrode site one by one in random order at 0.5 s intervals (2 Hz). The stimulation frequency was chosen to maximize the number of stimuli delivered while allowing population spike activity return to the prestimulus baseline. The same stimulation sequence was repeated 80 times, equivalent to a total of 2,560 stimuli delivered in each condition. Stimulation pulses were charge-balanced, biphasic waveforms (Merrill et al., 2005) that were initially varied with respect to cathodic-anodic temporal order and asymmetry of duration and amplitude. Preliminary tests in 4 animals with 5, 10, 20, and 40 μA stimulation currents showed that maximum response was afforded by 40 μA in agreement with other studies (Voigt and Kral, 2019; Sombeck et al., 2022; Yun et al., 2023). In all subsequent experiments a maximum stimulus current of 40 μA was used with 5:1 and 1:5 amplitude/phase duration ratios to maximize the number of similar trials within the same experimental time frame. The corresponding amplitudes and phase durations were 40 μA for 60 μs and 8 μA for 300 μs separated by 0 μA for 60 μs.

### Recording system and data analysis

**FIGURE 1:**
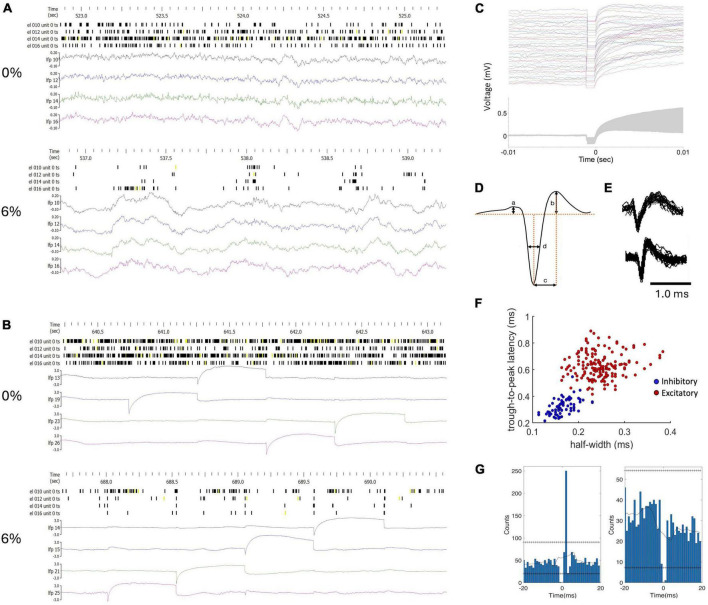
Classification of units and monosynaptic connectivity. **(A)** Example of raw spike trains (unclassified) and local field potentials (LFP) from four neurons in wakefulness (0%) and in desflurane anesthesia (6%). **(B)** Data from the same experiment during microstimulation. LFP channels were chosen to include stimulation events within the 2.5-second time window as shown. The amplitude scale of the LFP was adjusted to fit the stimulus artifact into range. **(C)** Stimulus artifacts at a selected electrode site. Top: 80 stimulus traces superimposed; Bottom: ± one standard deviation of signals around the mean (gray zone). **(D)** Features of spike waveform used for unit classification. **(E)** Examples of regular spiking excitatory neuron (top) and fast spiking inhibitory neuron (bottom). Several spike waveforms were aligned and superimposed. **(F)** Trough-to-peak time (b) and half amplitude width of the negative peak (c) provided separation of units into putative excitatory and inhibitory neurons with typical spike shapes. **(G)** Cross correlograms of neuron-pairs that indicate excitatory (left) and inhibitory (right) monosynaptic connections as an example. Horizontal lines of “+” symbols indicate high and low global confidence intervals obtained from spike-jittered average.

Extracellular potentials were recorded at all electrode sites except at the one being stimulated using the data acquisition system, Scout Processor and Nano2+Stim front end (Ripple Neuro, Salt Lake City, UT, USA), digitized at 30 kHz and band-pass filtered at 300–7,500 Hz for spike detection. Spike rasters and local field potential (LFP) traces in two conditions before and during microstimulation are shown in Figures 1A, B in one experiment as an example. The stimulus artifact was removed by template subtraction (Sombeck et al., 2022). As shown in Figure 1C, significant stimulus artifact at the non-stimulated electrodes was limited to 1 ms duration (Figure 1C). The amplitude of the subsequent slow potential was less than 1 mV and inconsequential for spike detection and classification following band-pass filtering. Electromyographic (EMG) artifacts due to motion were eliminated by rejecting data from all channels within ± 1 s of any signal exceeding 6 standard deviations of the mean. A total of 160 spiking neurons were recorded in 9 rats. Unit spikes were extracted and sorted using the software Spyking Circus (Yger et al., 2018). The number of recorded neurons in each condition in each animal is provided in Table 1. Neurons were classified off-line into putative excitatory or inhibitory type based on the half peak-width and trough-to-peak time of their spike waveform as illustrated in Figures 1D–F as done before (Lee et al., 2020).

**TABLE 1:** Number of recorded neurons.

	Desflurane			
Rat	0%	2.5%	4%	6%
1	16 (2)	15 (0)	16 (0)	13 (0)
2	23 (6)	21 (7)	19 (4)	11 (0)
3	22 (3)	24 (3)	15 (2)	21 (2)
4	18 (1)	17 (1)	16 (2)	7 (1)
5	22 (2)	22 (1)	22 (2)	17 (2)
6	17 (2)	15 (1)	14 (2)	2 (0)
7	25 (2)	21 (4)	17 (3)	21 (3)
8	21 (4)	22 (4)	21 (4)	15 (2)
9	18 (2)	16 (1)	17 (1)	9 (1)

Number of inhibitory neurons in parenthesis.

### Network analysis

Stimulus-related network connectivity was analyzed in two different ways. First, an effective connectivity map was constructed from all pairs of stimulus-neuron sites from neurons with a significant change in spike rate within 1–4 ms after stimulation. Spike counts from 20 trials at each stimulus site were averaged. Significant change was defined by a spike rate increase (or decrease) exceeding ± 95% confidence interval of the 200 ms pre-stimulus baseline. For each stimulation site, neurons with significantly increased firing rate were marked by a vector from the respective stimulation site. Pooling all vectors from all stimulation sites were then compiled in a directed graph. The resulting graph depicted the influence of microstimulation on all recorded neurons as a binary directed network.

Second, putative monosynaptic connections were determined from the short time lag spike cross-correlograms (CCGs) from spontaneous (pre-stimulus) and stimulation-induced activity as before (Vizuete et al., 2012; Kobayashi et al., 2019). Briefly, for each pair of neurons, CCGs were calculated at 1 ms time bins over an interval of ± 50 ms. To avoid false detection due to background fluctuations, the CCGs were also calculated from surrogate spike trains prepared by “jittering” the spike times within ± 5 ms, repeated 1,000 times to yield 1,000 surrogate data sets. The 99% confidence interval of the number of CCG counts in each time bin were obtained and their maximum and minimum were used as global thresholds. Putative excitatory or inhibitory monosynaptic connections between a pair of neurons recorded at different electrode sites were inferred by the presence of CCG peak exceeding (excitatory) or trough descending below (inhibitory) the respective global threshold within 1–4 ms time lag (Figure 1G). Each monosynaptic connection was represented by a vector between the respective electrode sites. Concatenation of such vectors were interpreted as putative polysynaptic paths. All such vectors were combined yielding a graph with its nodes being the electrode sites. The low-level structure of the graphs was analyzed by finding its 1st, 2nd and 3rd order connectivity motifs (Recanatesi et al., 2019). Motifs of each order were counted in each condition, pre- and post-stimulus. Motifs of the 2nd and 3rd order can have different variants, called unique motifs. Unique motifs are those different from all other motifs. Their number reflects motif diversity.

### Statistics

Within-group effects of microstimulation and anesthetic level were tested with ANOVA followed by Tukey Honestly Significant Difference post-hoc tests. All results are presented as means and standard deviation. Some of the analyses were limited to experiments that yielded the most neurons/spikes. Some of the analyses were limited to experiments that yielded the most neurons/spikes.

## Results

Simultaneous extracellular recording and electrical stimulation was performed in nine rats using 32-site microelectrode arrays in primary visual cortex at three levels of anesthesia and wakefulness. On average active neurons were found at 63% of the electrode sites which progressively dropped to 40% at 6% desflurane (*p* < 0.0001, ANOVA). Figure 1A presents an example of the firing pattern of neurons and local field potentials recorded at the same electrodes in wakefulness and anesthesia at baseline (without stimulation). As shown, the regular firing pattern in wakefulness converts to an intermittent pattern associated with slow wave activity in anesthesia.

**FIGURE 2:**
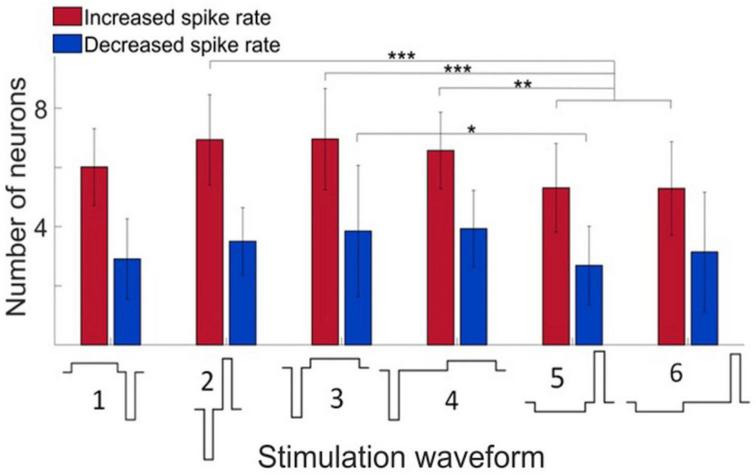
Effect of stimulus waveform on the number of neurons showing significant change in firing rate. Maximum stimulus current was 40 μA with 60 μs pulse width and charge balanced. Responses elicited from various stimulation sites were pooled. All data were obtained in wakefulness. Data are from 4 rats. Mean ± SD, *p < 0.05, **p < 0.01, ***p < 0.001.

Microstimulation was applied to each electrode site was then stimulated one by one in random order at 2 Hz repetition rate using charge-balanced biphasic pulses. We compared the effect of stimuli with different phase duration, amplitude, and temporal order on the firing response of neurons. In all stimuli, the maximum current was 40 μA with 60 μs pulse width and balanced by another phase with opposite polarity as illustrated in Figure 2. Putative excitatory and inhibitory neurons were not distinguished in this analysis. Cathodic-first charge-balanced stimuli activated more neurons than anodic-first charge-balanced stimuli, although this difference was small. All subsequent experiments were performed with asymmetric biphasic pulses with 40 μA maximum intensity and 60 μs pulse width, balanced with 8 μA intensity and 300 μs pulse width.

**FIGURE 3:**
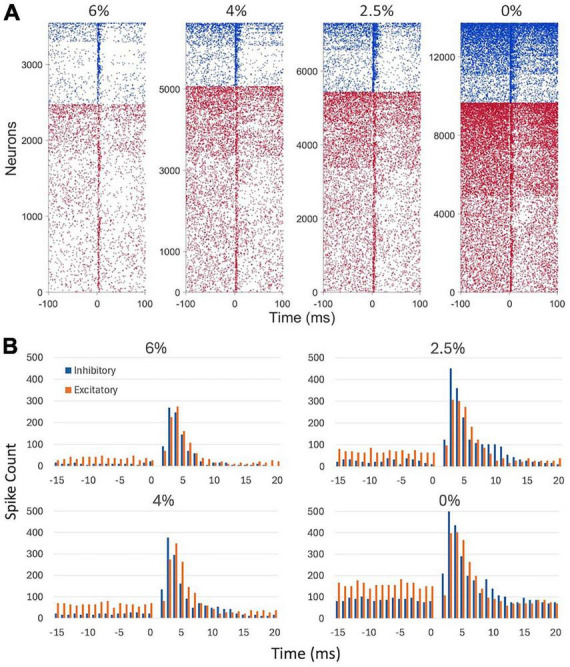
Effect of microstimulation on cortical spiking activity in four conditions. **(A)** Raster plots of putative inhibitory (blue) and excitatory (red) neuron firing. Inhaled concentration of the anesthetic desflurane is indicated on top. Each line corresponds to a neuron responding to a different trial, i.e., to stimulation at a different electrode site. Neurons with at least one spike within ± 100 ms of stimulation only were included. Neurons of both types were sorted vertically by their firing rate. Stimulus is at time 0. **(B)** Peristimulus histogram of spike counts. Spike data are absent in the 0–1 ms time bin due to stimulus artifact. Stimuli were charge-balanced, biphasic, asymmetric, cathodic-leading, pulses with 40 μA maximum intensity delivered at 2 Hz. Data are from 9 rats.

Figure 3 shows the time course of spike responses of putative excitatory and inhibitory neurons. Neurons were classified by their spike waveform features. Microstimulation generally caused an early increase, within 10 ms, followed by a prolonged (11–100 ms) decrease in neuron firing. Both excitatory and inhibitory neurons showed this behavior. Neurons responding to stimulation were scattered throughout the electrode array. Despite the smaller number of inhibitory neurons recorded, their total spike count was higher due to their generally higher average spike rate. The maximum spike rates attained post stimulus at 6, 4, 2, and 0% desflurane were 2.3, 4.0, 4.5, and 6.5 spikes/s, respectively, for excitatory neurons and 10.0, 15.9, 18.4, and 25.0 spikes/s, respectively, for inhibitory neurons.

**FIGURE 4:**
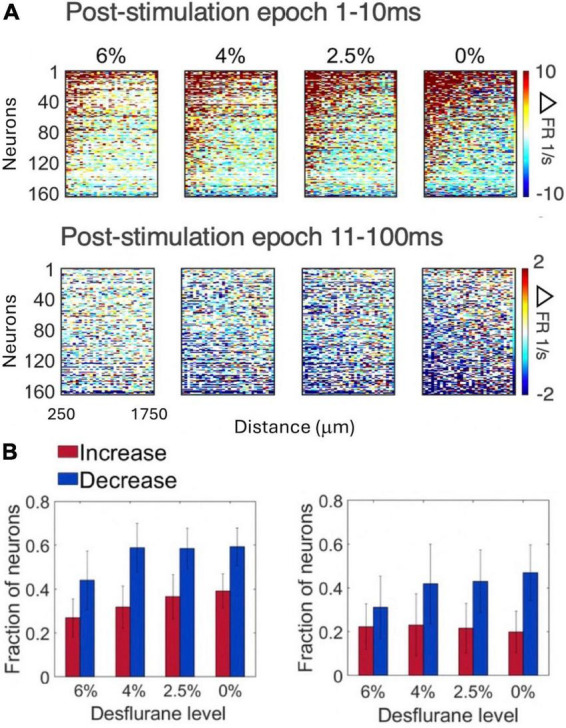
Change in firing rate as a function of distance from stimulation site in four conditions and two post-stimulus epochs. **(A)** Change in spike rate change of individual neurons. For each neuron, the 32 stimulation channels are arranged left to right in the order of increasing distance from stimulation site. Neurons are arranged top down from maximum increase (red) to maximum decrease (blue) of spike rate during the wakefulness (0%) and the same order is kept for the other three conditions. Desflurane concentrations are shown on top. Data are from 9 rats. **(B)** Fraction of neurons that produced statistically significant increase (red) or decrease (blue) in firing rate (1–10ms left, 11–100ms right). More neurons showed decrease than increase of firing rate with microstimulation (*p* < 0.0001, ANOVA), a difference qualitatively conserved across states. Stimuli were asymmetric, cathodic-leading, biphasic pulses with 40 μA maximum intensity delivered at 2 Hz.

Figure 4A compares the change in spike rate of each neuron as a function of distance from the stimulation site separately for the early (1–10 ms) and late (11–100 ms) response. The magnitude of spike rate increase decayed with the neuron’s distance from the stimulation site. In contrast, the magnitude of spike rate decrease was essentially independent of the distance from the stimulation site. This pattern was essentially conserved across all conditions. The number of neurons responding with a significant increase or decrease in spike rate to stimulation in the two epochs is illustrated in Figure 4B. The magnitudes of the early increase and late decrease tended to increase slightly at lighter levels of anesthesia and upon waking although these effects were not statistically significant. Approximately twice as many neurons decreased than increased their spike rate following microstimulation (*p* < 0.0001, ANOVA).

**FIGURE 5:**
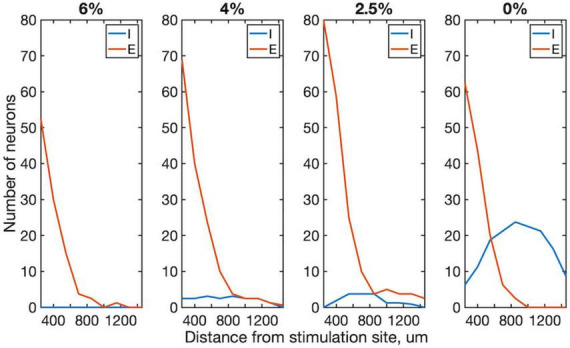
Effect of distance from stimulation site on excitatory and inhibitory neuron spiking within 1–4 ms post-stimulus interval in four conditions. The number of neurons whose firing rate exceeded 99% confidence interval of their prestimulus firing rate are plotted. Trials from all stimulus sites were combined. Data are from 4 rats. Desflurane concentrations is indicated on top. Stimuli were charge-balanced, asymmetric, biphasic, cathodic-leading pulses with 40 μA maximum intensity delivered at 2 Hz. *P* < 0.0001 (ANOVA and Tukey) for 0% vs. 2.5%, 4% and 6% for inhibitory; N.S. (ANOVA) for all excitatory neurons.

We examined more closely how the early response of excitatory vs. inhibitory neurons varied as a function their distance from the stimulation site. This analysis was limited to the first 4 ms compatible with putative monosynaptic effects. As Figure 5 shows, the number of excitatory neurons that significantly increased their firing rate decayed rapidly with distance from the stimulation site. This dependence was absent for inhibitory neurons suggesting that they were stimulated in a more distributed manner.

**FIGURE 6:**
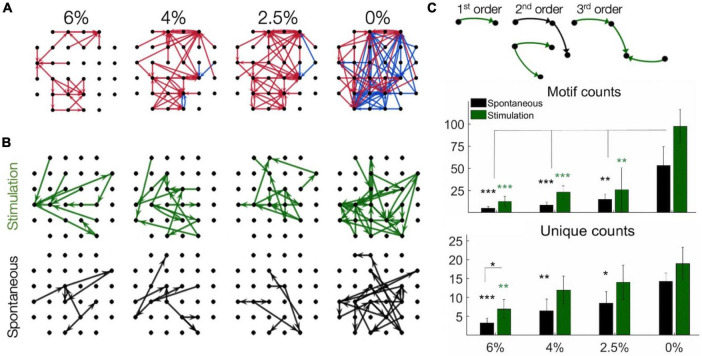
Effective connectivity of neurons determined by microsimulation. **(A)** Connectivity maps in one rat as an example. Arrows point from the site of stimulation to the site of significantly increased (red) or decreased (blue) spike rate within 1–4 ms post-stimulation (desflurane concentrations indicated on top). Desflurane concentrations is indicated on top. **(B)** Putative monosynaptic effective connectivity maps inferred from pairwise spike cross-correlograms pre-stimulus (spontaneous) and post-stimulus (stimulation). The graphs represent “multi-node” paths composed of pairwise connections. **(C)** Effect of anesthesia on the number of connectivity motifs as inferred from pairwise spike cross-correlograms before and after microstimulation. Examples of motifs of different order are illustrated on top. The counts of 1st, 2nd, and 3rd order motifs were combined. Unique counts reflect the diversity of distinct motif types. Desflurane concentration is on the horizontal axis. Data are from 4 rats. Mean ± SD, *p < 0.05; **p < 0.01; ***p < 0.001. Stimuli were charge-balanced, asymmetric, biphasic, cathodic-leading pulses with 40 μA maximum intensity delivered at 2 Hz.

We then examined the spatial pattern of stimulus-induced (effective) connectivity of neurons. This was done in two different ways. First, a directed graph was constructed from the vectors from each stimulation site to all neurons whose firing rate was either significantly increased or decreased by microstimulation (Figure 6A). The resulting connectivity map illustrates the overall influence of microstimulation on the spiking of all recorded neurons. Comparing different conditions, effective connectivity was sparse under anesthesia, suggesting reduced overall neuronal excitability. The total number of positive effects (neurons with increasing spike rates) was significantly greater as the anesthetic was withdrawn. Thus, the effective connectivity map indicated progressively stronger influence of stimulation at lighter levels of anesthesia and especially in wakefulness. Moreover, negative effects (neurons with decreasing spike rates) were almost absent at all levels of anesthesia. Second, we estimated effective connectivity from putative pair-wise monosynaptic connections as determined from spike cross-correlograms both pre- and post-stimulus. A directed graph was constructed by representing all monosynaptic connections as vectors between two respective electrode sites as graph nodes. Monosynaptic connections between neurons at different electrode sites only were considered (Figure 6B). When two or more monosynaptic connections occurred in series, their combination was interpreted as a putative polysynaptic path. As with the previous method, higher connectivity was evident in light anesthesia and wakefulness and more in the post-stimulus than pre-stimulus phase.

**FIGURE 7:**
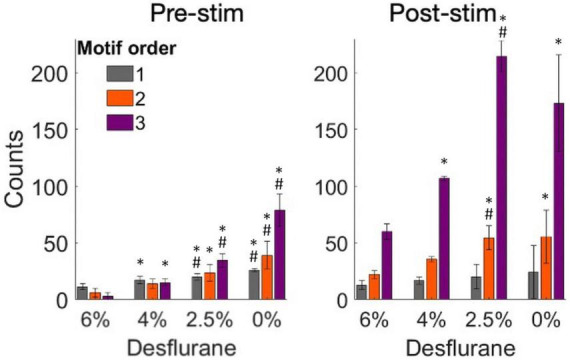
Change in the number of motifs of orders 1–3 as a function of experimental condition before and after microstimulation. Motifs were derived from pairwise spike cross-correlograms. Stimuli were charge-balanced, asymmetric, biphasic, cathodic-leading pulses with 40 μA maximum intensity delivered at 2 Hz. *p < 0.05 vs. at 6%, #*p* < 0.05 greater than at next higher desflurane concentration.

To further examine the structure of the effective connectivity map, we identified low-level functional motifs of the networks. A motif is a small subset of pairwise connections, representative of a unit of overall network connectivity. Pairwise connections are arranged in series or in diverging or converging patterns, leading to multiple variants of second, third and higher order motifs (Recanatesi et al., 2019). First order motifs consist of a single monosynaptic connection; second order motifs contain two connections; and third order motifs contain three connections. Second and third order motifs have multiple variants defined by their connectivity profile. The prevalence of motifs of increasing order, characterized by their relative frequency, reflects the complexity of the overall connectivity of the network and its change with stimulation and anesthetic condition. Figure 6C shows that microstimulation increased the total number of detected motifs, thus enhancing monosynaptic and putative polysynaptic connectivity at all levels of anesthesia although this effect did not reach significance at 0% desflurane in this comparison (for statistics see figure). Similar results were obtained for the number of unique motifs, suggesting that the diversity of motif variants also increased after microstimulation. In addition, both the total number of motifs and their diversity significantly increased as the anesthetic was withdrawn and reached maximum in wakefulness (see figure for statistics). Figure 7 compares the change in the number of motifs of different orders separately. A progressive increase in the number of all motifs with decreasing anesthetic concentration was evident both pre-stimulus and post-stimulus (*p* < 0.0001, ANOVA), except in post-stimulus first order motifs. In addition, microstimulation increased the number of motifs of all orders (*p* < 0.005, ANOVA) with the most dramatic effect seen in motif order 3 (for pairwise statistical comparison see figure).

## Discussion

The main goal of this work was to apply intracortical microstimulation to examine how anesthesia may alter effective connectivity of neurons in a local cortical circuit. Microstimulation was chosen as an effective means to modulate local neuronal activity and its propagation across adjacent and remote recording sites (Kwan and Dan, 2012; Kumar et al., 2013). Probing local circuitry by causal perturbation provides additional insight into neuronal connectivity underlying cortical information processing over that obtainable by recording spontaneous or physiological stimulus-related activity (Clark et al., 2011; Kumar et al., 2013; Cicmil and Krug, 2015; Sadeh and Clopath, 2020). Further, by investigating the concentration-dependent effect of anesthesia on intracortical stimulus-related connectivity has a unique potential to help better understand how neuronal networks function in altered states across experimentally controlled levels of consciousness (Arena et al., 2021).

Consistent with prior studies we found that intracortical microstimulation generally produced a biphasic neuronal response consisting of an early increase in firing within 10 ms followed by a transient suppression of 100 ms or so (Butovas and Schwarz, 2003; Sombeck et al., 2022; Yun et al., 2023). This biphasic pattern was conserved at all levels of anesthesia although the spike rate increases were lower during anesthesia than in the awake state. The exact duration of these phases was reported to vary with stimulus intensity (Yun et al., 2023), although this aspect was not investigated further here.

Also, as found before (McIntyre and Grill, 2000; Voigt and Kral, 2019), cathodic-leading charge-balanced stimulus waveforms were more effective in stimulating neuron firing than the reverse waveforms, although this difference in our preparation was quite small. Experimental and computational studies suggest that local cells are preferentially activated by cathodic-first asymmetrical charge-balanced biphasic stimulus waveforms, while fibers of passage is affected more by anodic-phase-first asymmetrical charge- balanced biphasic stimulus waveforms (Cogan, 2008; Voigt and Kral, 2019). However, it was also shown that stimulus polarity and asymmetry influence the probability and localization of neuron activation in an opposite manner (Stieger et al., 2022). Also, computational modeling suggests that the initial mechanism of activation is an antidromic propagation to the soma following axonal activation (Kumaravelu et al., 2022). Orthodromic propagation and synaptic transmission could then mediate the further propagation of activation. However, which of these mechanisms likely contributed to stimulation-evoked neuronal firing at the recording sites cannot be determined from the present data.

We also examined how anesthesia with desflurane modulated the stimulus response of putative excitatory vs. inhibitory neurons. Most inhalational anesthetic agents suppress spontaneous neuronal activity by facilitating inhibitory synaptic transmission and suppressing excitatory synaptic transmission (Franks, 2008) through a variety of actions on specific receptors, channels, and extrasynaptic and mitochondrial energy modulation, the relative extent of which vary with the particular anesthetic. In particular, desflurane potentiates GABA_A_ receptor-mediated inhibition, inhibits NMDA receptor mediated excitation, inhibit voltage-gated, and to a lesser degree, inward rectifying potassium channels and kainate receptors (Nishikawa and Harrison, 2003; Alkire et al., 2008). Despite the inhibitory potentiation observed *in vitro*, in the intact cortical circuit *in vivo*, the strength of monosynaptic excitatory connections is more suppressed than that of monosynaptic inhibitory connections (Vizuete et al., 2012). Although most anesthetics affect the cortical circuits directly (Hentschke et al., 2005), this is further modulated via subcortical centers including the brainstem, thalamus, and basal forebrain (Brown et al., 2010). Due to these complexities, the effect of intracortical microstimulation on excitatory vs. inhibitory neuron activity was difficult to predict *a priori*.

To investigate this question, the early neuron spike responses were examined in 1–4 ms post-stimulus period which limits the response to monosynaptic transmission (apart from possible electrotonic conduction). Transsynaptic responses to single-pulse cortical electrical stimulation were reported in a comparable time frame (Sombeck et al., 2022). Under anesthesia very few inhibitory neurons showed monosynaptic response, whereas excitatory neurons continued to respond to stimulation. The latter could be a result of disinhibition due to the suppression of inhibitory neurons, although this possibility requires further investigation. As the anesthetic was withdrawn, substantially more inhibitory neurons responded, suggesting a normalization of the E/I balance. The spatial distributions of stimulus-responding excitatory and inhibitory neurons were also different. Inhibitory neurons had a relatively wide distribution in wakefulness, consistent with their excitation by horizontal axonal projections that extend over 1 mm (Kisvarday, 1992). The suppression of their firing rate under anesthesia could be mediated by their dense connectivity by electrical coupling (Butovas et al., 2006). This does not seem to be the case for excitatory neurons, however. Indeed, the decay of the number of excitatory neurons with distance from stimulation site could be related to their relatively sparse connectivity (Overstreet et al., 2013). A similar spatial decay of connectivity over 1 mm was reported before (Eles et al., 2021). Alternatively, if the neuron responses to microstimulation were mediated by electrotonic excitation via field potentials, this effect would decay exponentially with distance from the stimulation site.

The main goal of this study was to determine how anesthesia altered microstimulation-induced effective connectivity of cortical neurons. The graded reduction in effective connectivity at increasing depth of anesthesia was consistent with an overall reduction in neuronal firing rate (Hudetz et al., 2009) impeding the ongoing neuronal interactions and the propagation of activity to remote sites. Moreover, the negative effects of microstimulation (neurons with decreasing spike rates) were virtually absent in anesthesia suggesting that they were at their minimum baseline activity. This is consistent with the direct effect of volatile anesthetics on cortical firing by augmenting inhibitory neurotransmission (Hentschke et al., 2005). The directed effective connectivity graphs derived from putative monosynaptic connections confirmed these findings, revealing sparser connectivity in anesthesia compared to the dense connectivity in wakefulness that was further augmented in the post-stimulus phase.

To gain further insight into network connectivity, we also analyzed the prevalence of simple network motifs in different conditions. Such motifs have been considered as building blocks of complex recurrent networks. The motifs’ statistical prevalence can determine overall network properties, such as dimensionality, code compression, computational flexibility, network input-output response, and memory (Hu et al., 2014; Recanatesi et al., 2019). In the rat visual cortex, recordings of layer 5 pyramidal neurons revealed the presence of small, strongly connected network units that determine overall network connectivity (Song et al., 2005). Our analysis revealed a sharp difference in the overall number of motifs between wakefulness and anesthesia consistent with the rapid collapse of network connectivity. This difference was most expressed in the number of 3rd order motifs in the pre-stimulus condition. Although we were not able to identify motifs of higher than 3rd order due to the limited size of the electrode array, it could be surmised by extrapolation that higher order motifs would be even more affected. Because the number of 1st order motifs (monosynaptic connections) was hardly affected by the anesthetic (especially post-stimulus), the increase in higher-order motifs was probably due to network reconfiguration rather than an overall change in pair-wise connectivity. The fact that microstimulation did not significantly increase the number of motifs in the awake condition could mean that they were already at their near maximum density.

Several limitations of the current study are recognized. First, due to the sparse sampling of neuronal network, effective connectivity maps represent a small subset of true neuronal connectivity. A complete reconstruction of the neuronal network would theoretically require stimulating all single neurons and their combinations of the entire neuron network (O’Doherty et al., 2012; Kumar et al., 2013; Sadeh and Clopath, 2020). Nevertheless, the observed change in graph density in our sample should reflect the general trend of the anesthetic effect. Second, neuronal connectivity as determined here could be in part from synaptic transmission and in part from electrical fields through a mixture of antidromic and orthodromic activation (Butovas and Schwarz, 2003). The use of cathodic-leading biphasic stimuli in the present experiments presumably favored orthodromic activation, although this could not be verified with extracellular recordings. In addition, the probability of evoking excitatory responses decayed with distance rapidly, which could be due to the increasing sparsity of synaptic terminals reached or the weakening of electrical field with distance. Although cross-correlograms with short time-lag likely reflect monosynaptic spike transmission probability between pairs of neurons, they can be confounded by common inputs with comparable temporal delay. Thus, the neuron connections derived here are approximate and both the monosynaptic connections and their combinations inferred as polysynaptic connections should be considered putative. Third, extracellular microstimulation affects not a single cell but an unknown population of cells and passing axons surrounding the stimulation electrode. These difficulties could be mitigated by neuronal redundancy, i.e., that many neighboring neurons often respond to similar stimulus features, that a relatively small group of cells can drive network activity in similar fashion (Yassin et al., 2010; Kwan and Dan, 2012; Emiliani et al., 2015; Bernardi et al., 2021), and that frequently recurring neuronal firing sequences represent a small fraction of all possible patterns (Luczak et al., 2007, 2009; Luczak and Maclean, 2012). Thus, effective connectivity as derived here is best interpreted as “embeddedness,” i.e., the influence of a local neuronal population on individual neurons at a distance. In fact, both experiments and computer simulations suggest that an estimation of neuronal embeddedness is a valid approach for network discovery (Vlachos et al., 2012; Kumar et al., 2013). Fourth, motifs are only a metric of the basic building blocks of the functional connectivity of a local neuron network. There are several other graph-based metrics; however, our graphs were relatively small and sparse to apply such methods to them. The use of larger electrode arrays may allow such an analysis in the future. Fifth, one could contend that the observed changes in connectivity may in part be accounted for by the anesthetic-induced decrease in overall firing rate. However, this is not thought to be the primary contributing factor of effective connectivity changes because stimulus-induced neuronal firing is often significantly elevated during anesthesia, especially at deep anesthetic levels (Hudetz and Imas, 2007). Sixth, the accuracy of electrode placement in layers 5/6 of the primary visual cortex was not histologically verified in this work. However, the exact location of the electrodes was not critical for the general conclusions of the study. Our former work supports that stereotaxic implantation provided sufficient targeting accuracy (Pillay et al., 2014). Finally, trial-to-trial variability due to ongoing activity limits the accuracy at which an invariant network can be reconstructed. Nevertheless, it is believed that the microstimulation-based perturbational approach augments the reproducibility of the mapped network by transiently suppressing background activity (Cheney et al., 2013). In the future, optical methods that allow more precise control of the number and type of directly stimulated neurons (Cicmil and Krug, 2015; Cavelli et al., 2023) including optogenetic stimulation with micro-light emitting diodes (LEDs) (Buzsaki et al., 2015; Wu et al., 2015; Kim et al., 2020) could be used. In addition, distinct temporal patterns of stimulation pulses could also be used to control the affected neuronal population (Eles et al., 2021) and one could also target the stimulation to specific cortical layers (Voigt et al., 2017; Urdaneta et al., 2021).

In summary, the present work demonstrates the anesthetic modulation of effective connectivity in rat visual cortex as probed by microstimulation *in vivo*. The suppression of network connectivity was associated with the preferential loss of higher order network motifs. The results are relevant to understanding the mechanisms of anesthetic-induced impairment of cortical information processing and by inference, loss of consciousness.

## Data availability statement

The raw data supporting the conclusions of this article will be made available by the author, without undue reservation.

## Ethics statement

The animal study was approved by the Institutional Animal Care and Use Committee (IACUC) of the University of Michigan. The study was conducted in accordance with the local legislation and institutional requirements.

## Author contributions

AH: Conceptualization, Data curation, Formal analysis, Funding acquisition, Methodology, Project administration, Resources, Supervision, Validation, Writing – original draft, Writing – review & editing.
